# Improving Cognitive Function Through High-Intensity Interval Training in Breast Cancer Patients Undergoing Chemotherapy: Protocol for a Randomized Controlled Trial

**DOI:** 10.2196/39740

**Published:** 2023-04-07

**Authors:** Rebekah Wilson, Dong-Woo Kang, Meghan Tahbaz, Mary Norris, Hajime Uno, Jennifer Ligibel, Jeffrey Guenette, Cameron Christopher, Christina Dieli-Conwright

**Affiliations:** 1 Division of Population Sciences Department of Medical Oncology Dana-Farber Cancer Institute Boston, MA United States; 2 Harvard Medical School Boston, MA United States; 3 Department of Medicine Morsani College of Medicine University of South Florida Tampa, FL United States; 4 Division of Breast Oncology Department of Medical Oncology Dana-Farber Cancer Institute Boston, MA United States; 5 Division of Neuroradiology Brigham and Women’s Hospital Dana-Farber Cancer Institute Boston, MA United States

**Keywords:** cognitive function, high-intensity interval training, exercise, breast cancer, chemotherapy, magnetic resonance imaging, MRI

## Abstract

**Background:**

More than 75% of patients with breast cancer treated with chemotherapy experience cognitive impairments (eg, memory and attention problems), commonly known as *chemo-brain*. Exercise, especially aerobic high-intensity interval training (HIIT), is associated with better cognitive function in healthy populations. However, clinical trials testing the impact of exercise interventions on chemotherapy-induced cognitive decline in patients with cancer are lacking, and the mechanisms through which exercise could improve cognitive function are unclear.

**Objective:**

The objective of the Improving Cognitive Function Through High-Intensity Interval Training in Breast Cancer Patients Undergoing Chemotherapy trial is to examine the effects of HIIT on cognitive function in patients with breast cancer undergoing chemotherapy.

**Methods:**

This 2-arm, single-center, pilot randomized controlled trial will randomize 50 patients with breast cancer undergoing chemotherapy to HIIT or attention control. The HIIT group will perform a supervised 16-week, thrice-weekly intervention, with each session including a 5-minute warm-up at 10% maximal power output (POmax), 10 sets of alternating 1-minute high-intensity (90% POmax) and 1-minute recovery (10% POmax) intervals, and a 5-minute cooldown (10% POmax). The attention control group will receive a stretching program with no exercise components and be asked to maintain their exercise levels for 16 weeks. The primary outcomes of the study are executive function and memory measured using the National Institutes of Health toolbox and resting-state connectivity and diffusion tensor imaging microstructure evaluated using magnetic resonance imaging. The secondary and tertiary outcomes include cardiorespiratory fitness, body composition, physical fitness, and psychosocial health. The study has been approved by the institutional review board of the Dana-Farber Cancer Institute (20-222).

**Results:**

The trial was funded in January 2019, with recruitment started in June 2021. As of May 2022, a total of 4 patients have consented and been randomized (n=2, 50% to exercise; n=1, 25% to control; and n=1, 25% nonrandomized). Trial completion is expected in January 2024.

**Conclusions:**

This first-of-its-kind study incorporates a novel exercise intervention (ie, HIIT) and comprehensive cognitive measures. If positive, our findings will establish the pilot efficacy of HIIT on chemotherapy-induced cognitive function in patients with breast cancer, providing the foundation for future larger phase-II and phase-III trials to confirm the findings and potentially establish HIIT as a standard of care for women undergoing chemotherapy for breast cancer.

**Trial Registration:**

ClinicalTrials.gov NCT04724499; https://clinicaltrials.gov/ct2/show/NCT04724499

**International Registered Report Identifier (IRRID):**

DERR1-10.2196/39740

## Introduction

### Background

Breast cancer is the most commonly diagnosed cancer among women in the United States, with approximately 280,000 new cases each year [1]. Advances in breast cancer treatment have increased breast cancer–specific and overall survival for women with early-stage breast cancer, but side effects of therapy affect quality of life and functional status during and after cancer treatment. In particular, up to 75% of patients with breast cancer treated with chemotherapy experience treatment-related cognitive impairments commonly referred to as *chemo-brain* [1,2]. Although *chemo-brain* is often associated with chemotherapy, similar cognitive changes may also present as a result of the cancer itself or conditions resulting from cancer treatment (eg, pain and infection). These alterations in memory, concentration, and other cognitive abilities may represent long-term effects that diminish the quality of life of patients with breast cancer and increase the risks of patient morbidity and mortality [3,4].

Although the neurological mechanisms underlying *chemo-brain* remain unresolved, previous studies have demonstrated an association between physical activity, such as walking or cycling, and enhanced cognitive function in patients who have undergone chemotherapy treatment [5-7]. High-intensity interval training (HIIT) in particular has been shown to improve cognitive flexibility, executive function, and psychological well-being in both older adults and youths [8-11]. This exercise modality consists of brief periods of intense exercise interspersed with recovery intervals and is a safe and feasible exercise approach for patients with breast cancer undergoing chemotherapy [12-14]. However, the impact of HIIT on cognitive function in women undergoing chemotherapy for breast cancer remains unknown.

### Objectives

The primary aim of the Improving Cognitive Function Through High-Intensity Interval Training in Breast Cancer Patients Undergoing Chemotherapy (CLARITY) trial is to examine the impact of a 16-week HIIT exercise intervention on cognitive function assessed using the Montreal Cognitive Assessment, the National Institutes of Health (NIH) toolbox, and quantitative magnetic resonance imaging (MRI) of the brain. We hypothesize that executive function and memory scores will be higher after exercise compared with baseline and the attention control (AC) group and that participants in the HIIT intervention will have enhanced functional brain connectivity and structural white matter tracts compared with baseline and the AC group. The secondary aims are to examine the effects of HIIT on body composition; biomarkers related to metabolic dysregulation, inflammation, and cardiotoxicity; physical fitness and function; and quality of life and psychosocial outcomes in patients diagnosed with early-stage breast cancer undergoing chemotherapy. We hypothesize that participants in the HIIT intervention will have improved body composition and psychosocial health compared with baseline and the AC group.

## Methods

### Study Design

**Figure 1:**
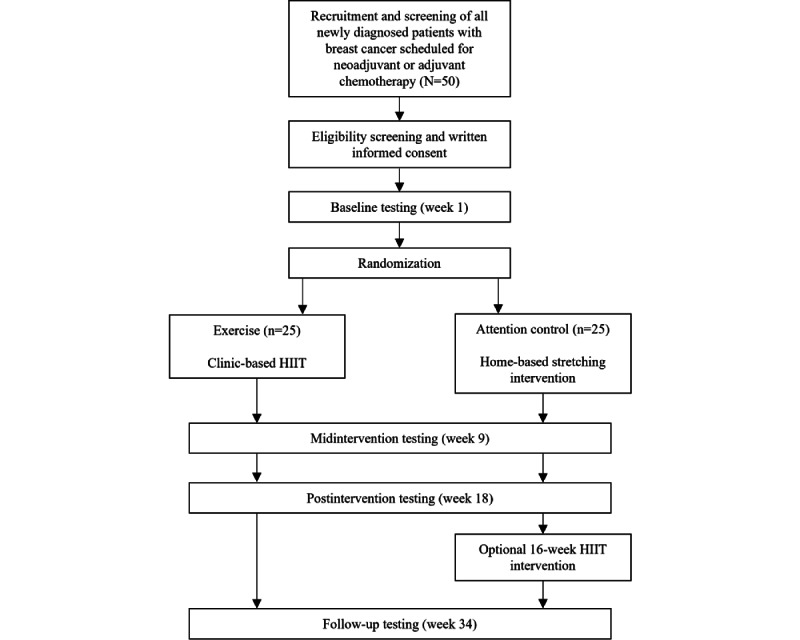
Study schema of the Improving Cognitive Function Through High-Intensity Interval Training in Breast Cancer Patients Undergoing Chemotherapy trial. HIIT: high-intensity interval training.

The CLARITY trial is a 2-arm randomized controlled trial underway at the Dana-Farber Cancer Institute (NCT04724499). Outcomes are measured at baseline (week 1), midintervention (week 9), after the intervention (week 18), and at follow-up (week 34; HIIT group only; Figure 1). A total of 50 women diagnosed with breast cancer who are chemotherapy-naïve are randomized either to a 16-week clinic-based HIIT intervention or an AC group who will be provided with a 16-week flexibility intervention. After the initial 16-week intervention period, the HIIT group completes a further 16-week follow-up period of self-directed exercise. The AC group does not complete the follow-up period and is instead offered the HIIT program. Only the AC participants who choose to partake in the HIIT program complete testing at the follow-up point (week 34).

### Participant Eligibility

Women are eligible if they are (1) newly diagnosed with breast cancer, (2) planning to receive neoadjuvant or adjuvant chemotherapy (regardless of duration and regimen of drug), (3) aged >18 years, (4) nonsmokers (within the previous 12 months), (5) inactive (defined as <60 minutes of structured exercise per week), (6) cleared by their clinicians for exercise, and (7) English-speaking. Exclusion criteria include (1) having a second malignancy or metastases; (2) pregnancy; (3) weight loss of >10% within the past 6 months; (4) having any uncontrolled comorbidity, condition, or contraindication that may prevent participation in high-intensity exercise; (5) having a pacemaker or any implantable device not safe for MRI; (6) claustrophobia; and (7) having a history of coronary heart or artery disease, chronic or acute congestive heart failure, or history of systolic or diastolic insufficiencies.

### Recruitment and Informed Consent

Participants are currently being recruited through clinician referral and screening of patient lists at the Dana-Farber Cancer Institute, Boston, Massachusetts, as well as by way of advertisements in patient waiting rooms, the Partners Rally Platform, and other relevant breast cancer community newsletters and servers. Pending accrual rate, potential additions to recruitment efforts to boost enrollment include offering travel compensation for testing visits for those who live outside the city and offering exercise sessions at additional satellite locations. All participants require clinician clearance before being formally screened via phone. Phone screening comprises 2 short questionnaires to determine eligibility: the Godin Leisure Time Questionnaire [15] to assist in determining the participant’s current exercise levels and the Physical Activity Readiness Questionnaire [16] to confirm the participants’ health status and how this relates to undertaking an exercise program. Upon confirmation of eligibility and interest, participants consent via wet or electronic signature.

### Ethics Approval

Approval of the trial protocol was obtained from the institutional review board at the Dana-Farber Cancer Institute (20-222).

### Randomization and Blinding

After baseline testing, participants are randomly allocated to either the HIIT or AC group using a 1:1 ratio and a permuted blocked design with varying block sizes where investigators are blinded to this process. The study biostatistician and coinvestigator (HU) prepared the randomization list before study start-up, after which the investigators access through a web-based application to conduct randomization and subsequently verbally inform the participants of their group allocation. Testers and participants are blinded to group allocation during baseline testing. Owing to the nature of the intervention and logistical limitations, participants and study staff cannot be blinded to the intervention allocation.

### Measurements

#### Overview

Testing is completed on 2 consecutive days at baseline, after the intervention, and at follow-up, with only 1 day required for testing at midintervention. At baseline, the first testing day may occur before the first dose of chemotherapy or within 10 days of the first infusion. The second day of testing is completed within 1 to 6 days of testing day 1 and may occur after the first cycle of chemotherapy. All measures are collected at all time points unless otherwise specified, and study staff members trained in exercise oncology will be supervising and leading all the tests unless otherwise specified (Table 1).

**Table 1:** SPIRIT (Standard Protocol Items: Recommendations for Interventional Trials) flow diagram for timeline of study visits for the Improving Cognitive Function Through High-Intensity Interval Training in Breast Cancer Patients Undergoing Chemotherapy trial.

Time point^a^			Week 0		Week 1-17		Week 9		Week 17 or 18^b^		Week 17-34		Week 34^c^
**Enrollment**													
	Eligibility screen	✓											
	Phone screen	✓											
	Informed consent	✓											
	Allocation	✓											
	Exercise screening	✓											
**Interventions**													
	HIIT^d^			✓									
	Attention control			✓						✓ (optional)			
**Assessments**													
	Executive function and memory	✓						✓				✓	
	MRI^e^	✓						✓				✓	
	Blood biomarkers	✓				✓		✓				✓	
	Anthropometrics	✓				✓ (no DEXA^f^)		✓				✓	
	Cardiorespiratory fitness	✓				✓		✓				✓	
	Muscle strength	✓				✓		✓				✓	
	Physical function	✓				✓		✓				✓	
	Psychosocial measures	✓				✓		✓				✓	
	Peripheral neuropathy	✓				✓		✓				✓	
	Lymphedema assessment	✓				✓		✓				✓	
	Shoulder function and range of motion	✓				✓		✓				✓	
	Dietary intake	✓				✓		✓				✓	
	ActiGraph	✓				✓		✓				✓	
	Activity logs			✓						✓			

^a^Week 0: enrollment and baseline assessment; week 1 to 17: intervention period; week 9: midpoint assessment; week 17 or 18: postintervention assessment; week 17 to 34: follow-up period; week 34: follow-up assessment.

^b^Depends on whether a participant has any exercise sessions to make up.

^c^Only for the high-intensity interval training group and the attention control participants who choose to complete the high-intensity interval training program during the follow-up period.

^d^HIIT: high-intensity interval training.

^e^MRI: magnetic resonance imaging.

^f^DEXA: dual-energy x-ray absorptiometry.

#### Primary Outcomes

##### Executive Function and Memory

To assess executive functioning and abilities, we use the NIH toolbox [17] and the Montreal Cognitive Assessment to assess global cognition; both tests are conducted at baseline, after the intervention, and at follow-up. From the NIH toolbox, the following tests are administered: Auditory Verbal Learning Test for immediate recall (memory), Picture Sequence Memory Test for episodic memory, Oral Reading Recognition for language, flanker task for executive function and attention, List Sorting Test for working memory, Oral Symbol Digit Test for processing speed, Dimensional Change Card Sort Test for executive function, and Pattern Comparison for processing speed. We also include an episodic memory composite that is derived from tests supported by the hippocampus and surrounding medial temporal lobe [18].

##### Resting-State Connectivity and Structural Diffusion Tension Imaging Connectivity

The MRI (Table 2) is conducted at baseline, after the intervention, and at follow-up by authorized MRI personnel. All imaging is performed on a single Siemens 3T Prisma MRI system with a 32-channel head coil. 3D T1 magnetization-prepared rapid acquisition with gradient echo imaging (1-mm isotropic voxels and 6-min, 38-s gradient time) is performed as the primary anatomic imaging sequence. Automated brain region segmentation is performed using the open-source FreeSurfer program (Martinos Center for Biomedical Imaging) [19] with manual correction [20]. Segmentation is used for primary volumetric analyses [21,22]. We examine the amygdala-hippocampus complex and thalamic and ventricular volumes and perform an exploratory comprehensive volumetric analysis. Multishell diffusion tensor imaging (DTI) is performed with 107 directions obtained in both the anteroposterior and posteroanterior directions (2-mm slice thickness and 6-min, 7-s gradient time each). DTI data are processed using the open-source 3D Slicer program (The Slicer Community) [23]. This technique enables traditional DTI analyses that are considered measures of cerebral microstructure and axonal and myelin pathology [24,25]. This technique also enables more novel analyses of free-water imaging [26], neurite orientation dispersion and density imaging [27], and diffusion kurtosis imaging [28], which can help evaluate for neuroinflammation and vasogenic and cytotoxic edema. Resting-state functional MRI (fMRI) is performed and analyzed using the Resting-State fMRI Data Analysis Toolkit (Hangzhou Normal University) [29]. We are examining the intrinsic network connectivity between the prefrontal cortex and hippocampus in addition to seed voxels in the posterior cingulate and precuneus of the default mode network [30].

**Table 2:** Magnetic resonance imaging parameters for the Improving Cognitive Function Through High-Intensity Interval Training in Breast Cancer Patients Undergoing Chemotherapy trial.

Sequence	Flip angle	Voxel size (mm^3^)	TE^a^ (ms)	TR^b^ (ms)	Acceleration	Extra notes
3D T1 MPRAGE^c^	8	0.8 × 0.8 × 0.8	2.22	2400	GRAPPA^d^ factor 2	Inversion time: 1000 ms
DTI^e^	78	1.5 × 1.5 × 1.5	89.20	3930	None	107 gradient directions both AP^f^ and PA^g^
rsfMRI^h^	52	2.0 × 2.0 × 2.0	37.00	800	None	AP direction

^a^TE: time to echo.

^b^TR: time to repetition.

^c^MPRAGE: magnetization-prepared rapid acquisition with gradient echo.

^d^GRAPPA: Generalized Autocalibrating Partially Parallel Acquisitions.

^e^DTI: diffusion tensor imaging.

^f^AP: anteroposterior.

^g^PA: posteroanterior.

^h^rsfMRI: resting-state functional magnetic resonance imaging.

#### Secondary Outcomes

##### Blood Biomarkers

A trained phlebotomist performs a fasting blood draw, obtaining both serum-separating tubes and EDTA samples. After being processed and aliquoted, the blood serum and plasma samples are stored in a −80 °C freezer for later batched analysis. Analysis of bloods through commercially available kits (Thermo Fisher Scientific) will be used for leptin, adiponectin, insulin, estradiol, matrix metalloproteinases, tissue inhibitors of metalloproteinases, interleukin-6, sex hormone–binding globulin, tumor necrosis factor-*α*, brain natriuretic peptide, N-terminal probrain natriuretic peptide, cardiac troponin I, and C-reactive protein. These biomarkers were selected based on their potential mechanistic role in how exercise may affect cognitive function [31-34].

##### Anthropometric Measures

Height (cm), weight (kg), and waist and hip circumference (cm) are assessed according to standard procedures [35], with height and weight used to calculate BMI (kg/m^2^). Body composition is assessed using bioelectrical impedance (Tanita 780), providing both appendicular and whole-body measures of fat and lean mass (kg).

##### Cardiorespiratory Fitness and Exercise Capacity

To assess maximal oxygen consumption (VO_2max_) and accurately prescribe the exercise intervention, a maximal cardiopulmonary exercise test is completed on a cycle ergometer (ErgoSelect 100; Ergoline) using an incremental ramp protocol [36]. Participants complete a 5-minute warm-up at a resistance of 40 W and then proceed into an incremental ramp protocol increasing 10 W every minute until volitional fatigue. Cadence is kept between 60 and 80 revolutions per minute. Heart rate (HR; FT4; Polar USA) and rate of perceived exertion (Borg scale 1-10) are recorded every minute and at the end of the test. Expired gas analysis (TrueOne 2400; ParvoMedics, Inc) is used to measure VO_2max_. The results of this test are used to calculate the target maximal power output (POmax) in watts for the high-intensity and recovery intervals of the HIIT program. In addition, the 6-minute Walk Test is used to estimate exercise capacity [37,38]. Participants will be instructed to walk as far as they can in 6 minutes up and down a 10-m walking course within a hallway. The distance achieved in 6 minutes will be measured in meters.

##### Muscular Strength

Muscular strength is assessed using an estimated 1-repetition maximum (RM) from 10-RM of leg press, leg extension, and leg curl (SportsArt and Matrix Fitness). Participants perform 1 to 2 warm-up sets of 6 to 8 repetitions. The participant proceeds to complete sets of 10 repetitions with increasing weights in each set until volitional fatigue is reached on the 10th repetition; a maximum of 3 sets are used to reach 10-RM. The weight of the participant’s 10-RM is recorded in kilograms and used to estimate 1-RM using validated equations [39,40]. Isometric handgrip strength is measured in the dominant upper limb (Camry Digital Hand Dynamometer). Participants perform 3 trials in a standing position with their arms down by their side, with the highest result used for analysis.

##### Physical Function

Physical function is assessed using the Short Physical Performance Battery (SPPB) [41], Timed Up and Go (TUG) [42], gait speed [43], Margaria stair climb [44], and sit-to-stand [45] tests. The SPPB comprises three sections: (1) balance with feet together, semitandem, and full tandem is held for up to 10 seconds with no support (only 1 attempt is given for each position, and the time in seconds to complete it is recorded); (2) usual gait speed over 4 m is timed, where the participant completes 2 attempts with the fastest time (seconds) recorded; and (3) chair stand, where the time (seconds) to complete 5 chair sit-to-stands is recorded, and only 1 attempt is given after a familiarization practice. Each section of the SPPB is given a score dictated by performance to then provide a summary score. The TUG test times how fast it takes a participant to stand up from a chair, walk around a cone placed 3 m away from the chair where they start, and end in a seated position. Participants attempt the TUG 3 times after a familiarization practice, where the average of the 3 trials is calculated. Gait speed is assessed over a 6-m flat surface where the time to walk the 6-m course at a usual and fast pace is recorded. In total, 2 attempts for each speed are completed. The Margaria stair climb is completed in a stairwell of 10 stairs. The participant walks or runs up the stairs as fast and safely as they can. The time (seconds) the participant takes from stair 3 to stair 9 is recorded; 3 attempts are given after a familiarization practice, where the average time of the 3 trials is calculated. Power (kg) is calculated from the stair climb using a validated equation [44]. The sit-to-stand test involves participants completing as many sit-to-stands as possible from a seated chair position to a standing position with full hip extension in 30 seconds; 1 attempt is given, with the number of full movements with correct technique recorded.

##### Psychosocial Measures

Health-related quality of life is assessed using the Functional Assessment of Cancer Therapy-Breast, which has an internal consistency coefficient of Cronbach *α*=.90 [46]. This is a 37-item questionnaire specifically designed for patients with breast cancer and measures the following 5 domains: physical, social, emotional, and functional well-being and a breast cancer subscale. Cancer-related fatigue is assessed using the Brief Fatigue Inventory, which has an internal consistency coefficient of Cronbach *α*=.96 [47]. This is a 6-item questionnaire that produces a global fatigue score by averaging all the items of the Brief Fatigue Inventory. Depressive symptoms are assessed using the Center for Epidemiologic Studies Depression Scale, which has an internal consistency coefficient of Cronbach *α*=.82 [48,49]. This is a 20-item questionnaire designed to measure a participant’s current level of depressive state using the following 6 scales reflecting major facets of depression: depressed mood, feelings of guilt and worthlessness, feelings of helplessness, psychomotor retardation, loss of appetite, and sleep disturbance. The State-Trait Anxiety Inventory is a 40-item questionnaire, which will be used to examine anxiety. The State-Trait Anxiety Inventory has an internal consistency coefficient of Cronbach *α*=.86 to .95 [50]. Sleep quality will be assessed using the Pittsburgh Sleep Quality Index, which has an internal consistency coefficient of Cronbach *α*=.83 [51,52]. This is a 19-item questionnaire evaluating 7 domains—subjective sleep quality, sleep latency, sleep duration, habitual sleep efficiency, sleep disturbances, use of sleep medication, and daytime dysfunction—and provides a global score. The impact that pain has on quality of life will be assessed using the Brief Pain Inventory-Short Form, which has an internal consistency coefficient of Cronbach *α*=.81 to .89 [53,54]. This is a 9-item questionnaire and has been validated for use in patients with breast cancer [53]. Finally, barriers to recruitment and exercise adherence are assessed using the Barriers to Recruitment Participation Questionnaire and Exercise Benefits/Barriers Scale, which have an internal consistency coefficient of Cronbach *α*=.93 [55,56]. The Barriers to Recruitment Participation Questionnaire is a 17-item questionnaire, and the Exercise Benefits/Barriers Scale is a 43-item questionnaire.

##### Peripheral Neuropathy

Peripheral neuropathy is measured using two tests: (1) the Semmes-Weinstein Monofilament Examination and (2) vibration testing using the on-off method [57-59]. For both tests, participants are seated in an upright position with their eyes closed and socks and shoes off. The Semmes-Weinstein Monofilament Examination is used to evaluate protective sensation at 2 sites—pad of the great toe (foot) and pad of the index finger (hand)—with the nylon monofilament applied 4 times to each site. The vibration sensation is evaluated by applying a tuning fork to the bony prominences of the great toe, thumb, and medial malleolus. Correct identification of the side of the body and on-off vibration sensation for the respective tests is recorded.

##### Lymphedema Assessment

Lymphedema is assessed using geometric arm volume calculations on both upper limbs [60]. We calculate arm volume using circumferential measurements taken at the following anatomic landmarks: axillary fold, halfway between the axillary fold and antecubital fossa, antecubital fossa, halfway between the antecubital fossa and wrist, and wrist. Circumferential measurements are taken with a constant-tension tape measure. Calculations for limb geometric volume are done using the frustum (truncated cone) volume, as described by Taylor et al [60]. A percentage difference between the lymphedema limb and the uninvolved limb is calculated to determine the amount of lymphedema. Lymphedema has been defined in recent literature as a >10% difference in volume calculation for the arm compared with the uninvolved upper extremity [60].

##### Shoulder Function and Range of Motion

Upper-body function is assessed using the Y Balance Test Kit (Move2Perform) [61]. Participants begin in a 3-point plank position on the toes or knees depending on ability. The tested shoulder is the supporting limb placed on the connecting block, the nontesting limb is placed on the medial block, and the feet are placed shoulder-width apart. From this position, the participant uses their nontesting limb to move each reach block one at a time in the medial, inferolateral, and superolateral directions in a controlled manner as far as they can and then return to the starting position. The test is performed 3 times on each side with a 30-second rest between trials. The reach distance in each direction is recorded, where the highest value in each direction is used to calculate an average composite score.

Bilateral upper-body strength and power are assessed through the seated medicine ball throw [62]. Participants are instructed to sit on the floor with their head, shoulders, and back against the wall and legs extended; legs may be bent if ability does not allow for full extension. To calculate a relative throwing distance, arm-reach distance is measured from the wall in meters. Using a 2-kg medicine ball covered in chalk, the participant performs a chest pass holding the ball with both hands and throwing it forward in a straight line as far as they can with head, shoulders, and back maintaining full contact with the wall. A measuring tape is used to measure the distance from the wall to the most proximal point of the chalk mark, with the participant’s reach distance subtracted to provide a final distance in meters. In total, 3 test trials will take place after a practice throw with a 1-minute rest between each trial. The average of the 3 trials will be calculated.

Shoulder function is assessed through the shoulder performance test using the following tasks: overhead reach, hand behind the head, and hand behind the back. All movements are completed in a standing position starting with the arms by the side. The time (seconds) taken to complete the respective tasks using the correct technique 20 times is recorded. Each task is repeated to the right and left.

Upper-body function is assessed using the closed kinetic chain upper-extremity stability test [63]. Participants begin in a 3-point plank position on the toes or knees, depending on ability, with hands 36 inches (91.4 cm) apart, shoulders perpendicular to hands, and feet hip-width apart. From this position, the dominant hand reaches across the body, touches the nondominant hand, and returns to the starting position. Subsequently, the same movement is performed by the nondominant hand. Participants are instructed to perform as many alternating touches as possible in 15 seconds while maintaining the correct push-up position. In total, 3 trials occur after familiarization, with 45 seconds of rest in between each trial.

Shoulder active range of motion (AROM) is measured on both upper limbs. Using a goniometer (EZ Read; Jamar), AROM is assessed in external rotation at 0°, external rotation at 90°, forward flexion, and abduction [64,65]. The participant stands for all tests except for external rotation at 90°, where the participant is lying on the ground with knees bent and feet hip-width apart. The starting position of the measured limb is dictated by the AROM being tested. The participant is asked to actively perform the required motion with the angle of movement recorded in degrees. In total, 3 active trials are performed after the tester passively moves the participant’s limb in the tested motion.

##### Physical Activity and Sedentary Behavior

Physical activity and sedentary behavior are assessed using the ActiGraph wGT3X-BT (ActiGraph LLC) at baseline, after the intervention, and at follow-up only. Patients wear the accelerometer on their hip for 7 consecutive days, excluding water-based activities and sleep. ActiLife software (ActiLife 6; ActiGraph LLC) is used to analyze the ActiGraph data. Only wake wear time is used, with a minimal data collection period set for inclusion in the analysis of 4 days of at least 600 minutes per day. Nonwear time is excluded from the analysis, defined as ≥90 minutes of consecutive zeros with a 2-minute spike tolerance [66]. Commonly used cutoff points among patients with cancer will be used to classify sedentary time (<100 counts per minute), light physical activity (100-1951 counts per minute), and moderate to vigorous physical activity (≥1952 counts per minute) [67-69].

#### Covariates

##### Participant Characteristics

Participant demographic and medical history data are collected at baseline with the administration of a study-tailored questionnaire. In addition, patients complete the Socioeconomic Status Questionnaire to determine economic demographics, education level, and socioeconomic status [70]. Changes in demographic and medical history data (eg, medical conditions, medication, and cancer treatment) will be monitored throughout the study period and confirmed at each testing time point.

##### Dietary Intake

Recent dietary patterns are assessed using the automated self-administered 24-hour dietary assessment tool [71]. Participants complete 3 assessments at home on 2 weekdays and 1 weekend day, recording all food and drinks consumed during the previous 24-hour period.

##### Physical Activity Logs

The AC group completes a daily activity log throughout the 16-week intervention. During the supervised exercise sessions, the HIIT group is asked about their physical activity completed outside the supervised sessions over the previous week. In addition, they complete daily activity logs during the follow-up period. In the activity logs, the participants are asked to record the type of exercise performed, the time spent performing the exercise, the intensity of the exercise bout (rate of perceived exertion [Borg scale 1-10] or average HR for the HIIT group if they are wearing an HR monitor), and the day of the week in which it was completed.

### Intervention

#### HIIT Group

Participants randomized to this group partake in a clinic-based HIIT program using a cycle ergometer 3 days per week for 16 weeks under the supervision of a trained exercise oncology specialist. Before beginning the exercise session, participants undergo a screening procedure to ensure that they are fit to participate. Participants are permitted to participate in the exercise session if they present with normal body temperature (≤100 °F or ≤37.8 °C), resting HR (absence of bradycardia, <60 beats per minute; or tachycardia, >100 beats per minute), and resting blood pressure (≤200/120 mm Hg). In addition, participants complete the Exercise-Induced Feeling Inventory [72] before and after the HIIT session to assess the participants’ feeling state changes after exercise and self-motivation to adhere to an aerobic exercise regimen. Participants also complete the Therapy-Related Symptom Checklist [73], a 25-item questionnaire, to assess and identify patient-reported symptom occurrence and severity.

**Figure 2:**
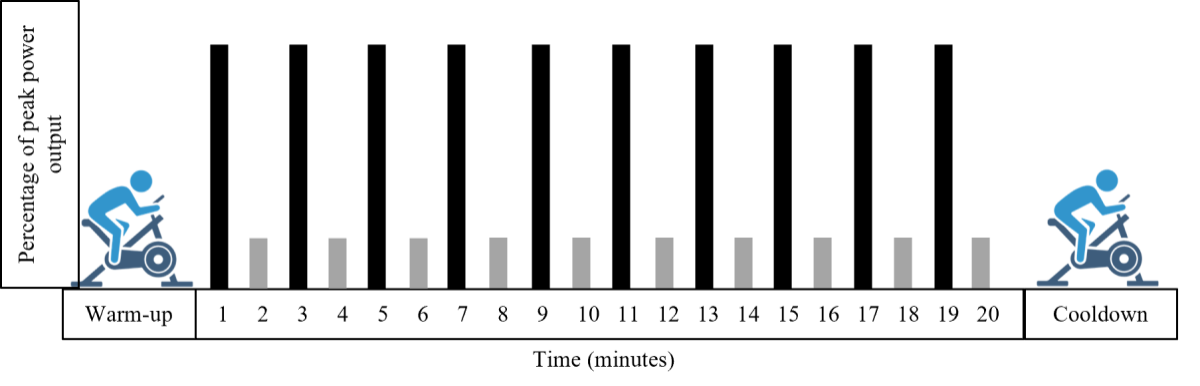
Example of a high-intensity interval training session and the high and low intensities performed.

The cycle-based HIIT exercise session consists of a 5-minute warm-up period at 10% of POmax (watts; calculated from the cardiopulmonary exercise test) followed by a 20-minute stimulus period consisting of alternating high-intensity interval bouts at 90% POmax (based on individual fitness level) with a recovery bout at 10% at a 1 minute:1 minute (work:recovery) ratio (Figure 2). Following the stimulus period, participants complete a 5-minute cooldown at 10% POmax (watts). An HR monitor wristwatch and transmitter are used to measure HR before exercise and at every minute during warm-up, exercise, and cooldown. Exercise sessions are completed with at least one full day of rest in between each session and are not to be completed on days when the participant has a chemotherapy infusion. Weekly goals will be set by the study staff for each participant based on their symptoms and how they feel overall, allowing for tailored sessions to accommodate individual fitness ability and treatment burden.

**Figure 3:**
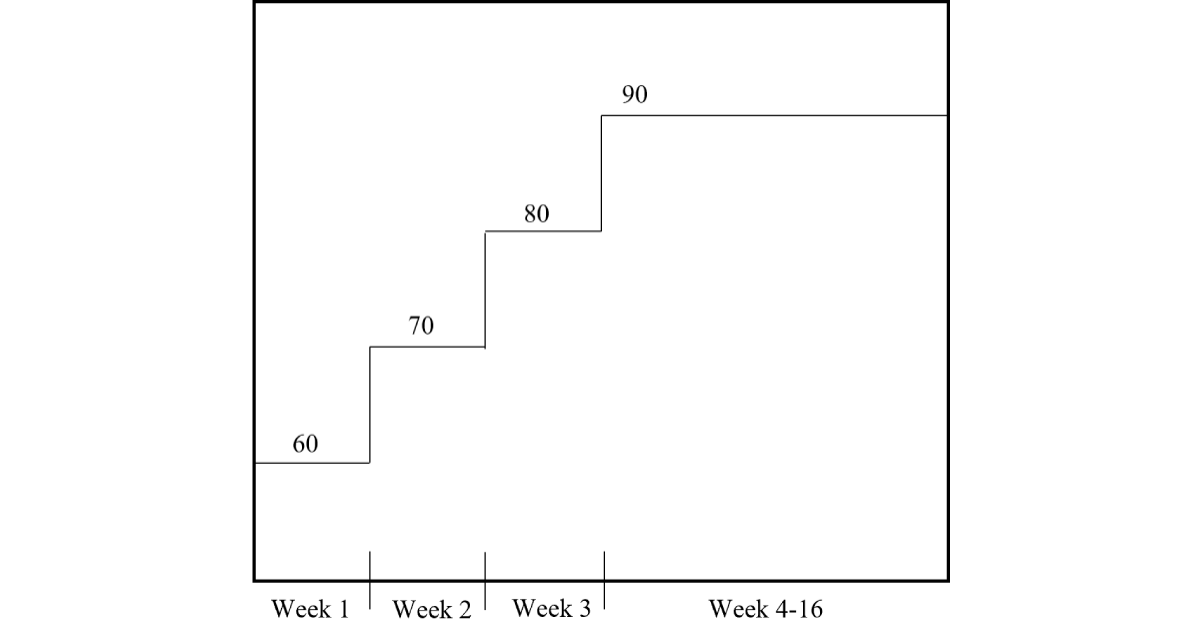
High-intensity bout progression over the course of the 16-week high-intensity interval training intervention.

Exercise intensity is gradually increased within the first 4 weeks of the intervention, whereby the participants begin the high-intensity interval bouts at 60% POmax, increase by 10% each week until 90% is reached, and then maintain at 90% for the remainder of the intervention (Figure 3). If a participant is unable to achieve compliance with the prescribed HIIT program, a moderate-intensity interval training (MIIT) program is substituted as an alternative exercise prescription. A single session is considered noncompliant if the number of high-intensity bouts achieving the target power output is <80% (eg, if ≥3 high-intensity bouts do not reach the prescribed power output). If 3 concurrent exercise sessions are reported as noncompliant, the participant is offered the MIIT program instead. Within the MIIT program, participants continue to complete intervals at a 1 minute:1 minute (work:recovery) ratio; however, the high-intensity bout is replaced with a moderate-intensity power output target. The intensity of the moderate interval is calculated as a percentage of the individual’s estimated VO_2max_ and will be selected according to the highest intensity that the participant may tolerate or complete. After the aerobic exercise session, the participants will perform 1 set of 3 to 4 static stretching exercises held for 30 seconds.

#### AC Group

Participants randomized to this group perform a home-based flexibility intervention 3 days per week for 16 weeks. The stretching protocol consists of the same stretching exercises as in the HIIT program. As flexibility exercises are low-intensity, low-impact, and low-volume, minimal caloric expenditure is expected. To increase compliance [74] and aid in the standardization of the home-based stretching [75], participants are provided with an instructional booklet of the flexibility exercises. Participants are asked to enter these stretching bouts into the daily activity log in addition to the other self-directed exercise sessions performed. Participants in the AC group are offered to participate in the same experimental exercise condition (HIIT) after the first 16-week study period; if they do not choose to participate in the HIIT program, they are not required to complete follow-up testing and are finished with the study after the first 16 weeks. The AC participants who choose to take part in the HIIT protocol after the initial 16 weeks return at the follow-up testing time point for final testing, which serves as post-HIIT testing.

#### Intervention Adherence

Participant adherence to the HIIT program is measured using (1) percentage and number of prescribed sessions attended (participants must attend ≥80% of the 48 sessions to be considered compliant with the HIIT program; eg, ≥38 sessions) and (2) average minutes of exercise per week (participants must complete ≥80% of the prescribed 90 minutes per week; eg, ≥72 minutes per week). To support adherence throughout the intervention, flexible hours are offered for exercise sessions, including before or after normal working hours and on weekends. In addition, all participants are given an additional 2 weeks beyond the 16-week intervention period to make up for any exercise sessions missed because of illness, work, or travel. Participants are provided with monetary compensation for each MRI completed and parking validation for every visit to the Dana-Farber Cancer Institute.

### Adverse Events

Any expected and unexpected adverse events are or will be reported to the principal investigator, who then subsequently reports to the institutional review board of the Dana-Farber Cancer Institute. Serious events must be reported within 24 hours of occurrence or finding out about the event. All adverse events, both serious and nonserious, and deaths that are encountered from the initiation of the study intervention, throughout the study, and within 30 days of the last study intervention are followed to their resolution or until the principal investigator assesses them as stable or determines the event to be irreversible or the participant is lost to follow-up. The presence and resolution of adverse events are documented on the appropriate case report form and recorded in the participant’s medical record.

### Data Monitoring and Management

Data are monitored internally within the Dana-Farber Cancer Institute for timeliness of submission, completeness, and adherence to protocol requirements. Monitoring begins at the time of participant registration and will continue during protocol performance and completion. The study team collects, manages, and performs quality checks of the data. Potential audits or inspections may be conducted by the principal investigator or their designated representatives. All data are stored on a secure network drive using REDCap (Research Electronic Data Capture; Vanderbilt University), a Health Insurance Portability and Accountability Act–compliant web-based application hosted by Partners HealthCare Research Computing, Enterprise Research Infrastructure & Services, on password-protected computers. Any hard-copy data are stored in locked filing cabinets in card-access facilities. The results of this study will be presented in publication, conference, and invited speaker formats.

### Sample Size Calculation

We will enroll 50 patients with breast cancer (n=25, 50% per group) in this study. We estimate that the dropout rate will at most be 20%. Assuming a 20% dropout, data from 40 (n=20, 50% per group) patients will be available for the analyses. With this sample size, we will be able to estimate within-group means for our outcome variables with a 95% CI of –0.47 to +0.47 SD units and mean differences between groups with a 95% CI of –0.64 to +0.64 SD units. Although the primary objective of the analysis for this pilot study is not to detect a between-group difference but to estimate the effect size for designing a subsequent confirmatory study, we also calculated a detectable effect size for the between-group comparison for reference. This sample size offers 80% power to detect a between-group difference at a .05 2-sided *α* level when the difference is 0.91 SD units.

### Statistical Analysis

Analyses will reflect the trial hypotheses that involve comparisons of HIIT and AC. Initial descriptive analyses will evaluate baseline comparability between the 2 intervention groups using the 2-sample, 2-tailed *t* test or 2-sample Wilcoxon test for continuous variables and the chi-square test or Fisher exact test for categorical variables. The primary analysis variables will be changed from baseline to week 16 of neuroimaging and cognitive function measures. Summary statistics (mean, SD, median, IQR, minimum, and maximum) will be used to describe the distribution of the variables. The mean and median will be reported along with the corresponding 95% CIs. Paired tests will be performed for these continuous outcomes for each group to assess whether there is a significant difference between baseline and week 16 in each group. For between-group comparison, a 2-sample *t* test will be performed. Analysis of covariance will also be used to assess the intervention effect on those continuous outcomes (changes from baseline to week 16), adjusting for patient baseline characteristics and other clinical covariates, including any participant who is required to switch to MIIT. Furthermore, those who are transferred to the MIIT group will be included in the HIIT group in the primary analysis based on the intention-to-treat principle. As an exploratory analysis, we will identify baseline factors of the patients who are likely to be transferred to the MIIT group. We will then exclude those who are transferred to the MIIT group and compare the HIIT and AC groups adjusting for factors that affect the transfer from HIIT to MIIT. Chi-square tests will be used for group comparison of binary outcomes (eg, the occurrence of adverse events). The magnitude of the intervention effect on binary outcomes will be summarized using odds ratio and the corresponding 95% CI. For each analysis, a 2-sided *P* value of <.05 will be considered statistically significant. As this is a pilot study, we will not adjust for multiple testing. Linear mixed-effects models will be used to estimate the trajectories of those repeatedly measured continuous outcomes across the intervention to assess group differences and explore the impact of potential prognostic factors (eg, body weight, age, fat mass, race, and menopausal status) on outcomes. Linear mixed-effects models will also be used for exploratory analysis of potential neuroimaging biomarkers. The primary analysis will include all completed outcome assessments (regardless of whether the woman stayed on the intervention), inviting those who drop out of the intervention to return for outcome assessments. The only missing data would be of those who drop out of the intervention and do not return for outcome assessments. Preliminary analyses will compare women who do and do not contribute to this analysis of baseline characteristics. A sensitivity analysis will include multiple imputations of all missing outcome data, including those patients who have missing assessments.

## Results

The CLARITY study was funded in January 2019 (American Institute of Cancer Research); however, recruitment was delayed until June 2021 as a result of the COVID-19 pandemic. As of May 2022, we have enrolled 4 patients in the study (n=2, 50% to exercise and n=1, 25% to control), with the fourth patient providing consent but withdrawing before baseline testing owing to chemotherapy not being pursued. In total, 50% (1/2) of the exercising patients also withdrew at week 6 of the intervention because of changes in time commitment. Recruitment is due to be completed in January 2024, with the primary results expected to be published in August 2024.

## Discussion

### Expected Findings

The CLARITY trial is the first randomized controlled trial to examine the impact of HIIT on cognitive function in patients with breast cancer undergoing chemotherapy. We hypothesize that undertaking HIIT will lead to improved executive function and memory scores and advance the current scientific knowledge regarding exercise and chemotherapy-induced brain function by incorporating (1) HIIT as an effective intervention that can enhance cognitive function; (2) multidimensional yet novel approaches to assess the effects of exercise on *chemo-brain*, such as fMRI, DTI, and the NIH toolbox; and (3) important secondary outcomes to provide a comprehensive understanding of the links between exercise and chemotherapy, including body composition, biomarkers related to metabolism, inflammation, cardiotoxicity, physical fitness and function, and quality of life and psychosocial outcomes.

Most patients with breast cancer experience cognitive decline during chemotherapy (up to 75%) [76,77], which may linger after treatment and even increase the risk of morbidity and mortality [78]. The onset of chemotherapy-induced cognitive decline has been an important clinical issue over the last few decades; however, the underlying mechanisms and potential interventions that can address cancer-related cognitive decline are still largely under investigation [3]. A promising nonpharmacological intervention is to engage in active physical movement, and a body of evidence supports the association between higher physical activity levels and improved cognitive function in patients with cancer [79]. A recent nationwide cohort study of 580 patients with breast cancer and 363 age-matched controls reported that patients who met the physical activity guidelines (ie, 150 minutes per week of moderate to vigorous physical activity) during chemotherapy showed significantly better self-reported cognitive function than those who did not meet the guidelines [80]. Nevertheless, the impact of exercise interventions on cognitive function in women receiving chemotherapy for breast cancer is not known. Previous exercise oncology trials have reported the preliminary benefits of exercise for cognitive function, but the overall quality of studies has been poor [79], warranting rigorously designed exercise clinical trials to explore the efficacy and mechanisms of exercise and cognition outcomes in patients with cancer undergoing chemotherapy treatment.

There are potential biological mechanisms to explain the benefits of exercise for chemotherapy-induced cognitive function. Exercise induces the expression of neurotrophic and neuroprotective markers, including brain-derived neurotrophic factor [81] and vascular endothelial growth factor [82], which contribute to “neurogenesis” in certain brain areas (eg, the hippocampus), leading to an enhancement of memory space and consolidation [83]. This mechanism is particularly plausible given that the hippocampus can be degenerated by toxic chemotherapy agents [84,85], whereas exercise has shown to not only improve hippocampus-dependent cognitive capacities but also increase the volume of the hippocampus [86-89]. Furthermore, neurological degeneration is significantly influenced by inflammatory markers [90], and exercise training can create a systemic anti-inflammatory environment to fight against and restore chemotherapy-induced cognitive decline [91-93].

Prior work has suggested that HIIT has significant potential to address chemotherapy-induced cognitive decline. For example, HIIT significantly improved cognitive responsiveness when compared with continuous moderate aerobic exercise in healthy young adults [94]. HIIT resulted in shorter reaction times and better response accuracy than continuous moderate-intensity exercise, suggesting that HIIT-induced neural adaptations redistribute neural resources to efficiently accomplish goal-directed behavior during cognitively demanding tasks. The observed decrease in P3 amplitude from fMRI following HIIT along with enhanced behavioral outcomes may indicate the implementation of greater efficiency in neuroelectric function. In addition, HIIT has successfully improved cognitive performance in patients with multiple sclerosis with reduced cognitive performance [95] and cancer-related fatigue in patients with breast cancer [14]. Furthermore, HIIT has shown superior effects on cognitive function (ie, verbal memory and executive functions) when compared with continuous moderate-intensity exercise in older adults [8] and individuals with Parkinson disease [96]. Therefore, HIIT may have the unique capacity to improve cognitive function in patients undergoing breast cancer chemotherapy.

Of importance, HIIT is a clinically proven, safe, and cost-effective intervention [97,98]. A recent study investigating HIIT in patients with heart failure with reduced ejection fraction revealed that the number of adverse events was not statistically different between the HIIT and moderate-intensity exercise group [99]. Furthermore, lower volumes of HIIT compared with moderate continuous-intensity exercise elicit better cardiovascular and physical fitness outcomes such as VO_2max_; therefore, it is a time-efficient exercise option that promotes greater gains in cardiovascular outcomes [98]. During chemotherapy, the optimal type, timing, and intensity of exercise interventions are unclear. As such, the exercise protocols examined during chemotherapy in previous breast cancer studies are varied, with many using aerobic interventions with common exercise prescriptions, including 3 to 5 times per week of 30-75 minutes of moderate-intensity (40%-70% predicted maximum HR) continuous exercise rather than the on-off interval strategy of HIIT [100]. Given the potential barrier of compliance to regular exercise participation during chemotherapy in patients with breast cancer, HIIT is an intriguing option for increasing cardiovascular benefits with shorter exercise duration, which serves as an efficient alternative to traditional endurance-based exercise training [12].

The strengths of our study include the randomized controlled design, comprehensive set of cognitive and biomarker measures, and the inclusion of delayed exercise for the AC group to enhance the retention rate. We also acknowledge that our study has several weaknesses. There is potential recruitment bias, such as participants in our study who are already interested in exercise and, therefore, relatively more fit than other patients with breast cancer undergoing chemotherapy who are not keen to participate in such exercise studies. In addition, the clinic-based setting of our intervention may not be reproducible in other intervention settings, such as home-, remote-, or community-based exercise programs. Upon publication of positive findings of this pilot study, future studies would be beneficial to understand effective dissemination strategies within the standard of care and the best timing of implementation of HIIT for patients with breast cancer undergoing chemotherapy to prevent chemotherapy-induced cognitive decline.

### Conclusions

The CLARITY trial is a first-of-its-kind study that incorporates a novel approach using a high-intensity exercise intervention (ie, HIIT), comprehensive cognitive measures (ie, quantitative fMRI and the NIH toolbox), and important secondary outcomes (ie, biomarkers, physical fitness and function, and psychosocial health). This study is anticipated to contribute to the current evidence on the role of HIIT in managing cognitive function and various patient-reported outcomes in patients with breast cancer undergoing chemotherapy and will establish the pilot efficacy to inform future larger phase-II and phase-III trials to confirm the findings.

## Abbreviations

AC: attention control
AROM: active range of motion
CLARITY: Improving Cognitive Function Through High-Intensity Interval Training in Breast Cancer Patients Undergoing Chemotherapy
DTI: diffusion tensor imaging
fMRI: functional magnetic resonance imaging
HIIT: high-intensity interval training
HR: heart rate
MIIT: moderate-intensity interval training
MRI: magnetic resonance imaging
NIH: National Institutes of Health
POmax: maximal power output
REDCap: Research Electronic Data Capture
RM: repetition maximum
SPPB: Short Physical Performance Battery
TUG: Timed Up and Go
VO2max: maximal oxygen consumption

## Data Availability

Data sharing is not applicable to this paper as no data sets were generated or analyzed during this study.
